# The Adult Epiglottitis Enigma: A Case Report

**DOI:** 10.7759/cureus.49984

**Published:** 2023-12-05

**Authors:** Mitanshi Luhana, Jumana Karim

**Affiliations:** 1 Otolaryngology - Head and Neck Surgery, Wrightington, Wigan and Leigh NHS Foundation Trust, Wigan, GBR; 2 General Surgery, Wrightington, Wigan and Leigh NHS Foundation Trust, Wigan, GBR

**Keywords:** airway emergency, acute epiglottitis, stridor, upper airway diseases, adult epiglottitis

## Abstract

Adult epiglottitis, once primarily associated with pediatric populations, has emerged as a distinctive clinical entity with potentially life-threatening implications. This condition is characterized by inflammation and swelling of the epiglottis, presenting initially as a seemingly benign sore throat and dysphagia but progressing rapidly to more severe symptoms such as drooling, severe odynophagia, hoarse voice, and acute upper airway obstruction. Timely diagnosis and intervention are paramount, as delayed presentation can result in fatal outcomes even in adults. The cornerstone of treatment involves securing the airway, providing supplemental oxygen, and administering intravenous antibiotics. In this report, we present a case of adult epiglottitis in a 20-year-old individual, discussing the clinical presentation, diagnostic considerations, and the essential components of its management. Recognition of adult epiglottitis as a distinct clinical entity is crucial for healthcare practitioners to ensure prompt intervention and optimize patient outcomes.

## Introduction

Epiglottitis, a potentially life-threatening condition, has long been recognized primarily as a pediatric concern, but its prevalence in adults is an important clinical consideration. Characterized by the inflammation of the epiglottis, a small, cartilaginous flap located at the base of the tongue prevents food from entering the trachea- this condition can lead to severe respiratory distress and requires immediate medical attention.

Historically, the incidence of epiglottitis in children has significantly declined due to the widespread implementation of the Haemophilus influenzae type b (Hib) vaccine; however, adult cases have concurrently displayed a relative increase. This shift underscores a different epidemiological pattern and necessitates awareness among clinicians regarding its presentation in the adult population [1]. Adult epiglottitis, while rare, tends to present more insidiously than in children and can be precipitated by various factors, including other bacterial agents, viral infections, and even traumatic or irritant causes [2].

Clinical presentation in adults can be variable, ranging from sore throat, odynophagia, and muffled voice to severe cases featuring stridor, drooling, and respiratory distress. Early recognition and management, typically securing the airway and antibiotic therapy, are crucial to prevent rapid deterioration [3].

Imaging studies, particularly neck soft tissue radiography or CT scans, can aid in diagnosis, though direct visualization via laryngoscopy remains the definitive diagnostic approach. However, this should be done cautiously in a controlled environment due to the risk of precipitating complete airway obstruction [4].

In conclusion, adult epiglottitis, though less common than its pediatric counterpart, is a critical diagnosis that must not be overlooked. The evolution of its epidemiology and presentation necessitates an updated understanding and approach in the adult patient, emphasizing swift recognition and management to ensure optimal outcomes.

## Case presentation

A 20-year-old male presented to our emergency department with a 2-day history of sore throat and difficulty in swallowing. He also complained of fever, body aches, and a hoarse voice. Neck movements were painful, especially on flexion of the neck. He denied any shortness of breath, history of nausea or vomiting, and chest pain. He hadn't been able to sleep for two nights and was having hallucinations with sensitivity to light. He had no significant past medical history and no known drug allergies.

**Figure 1:**
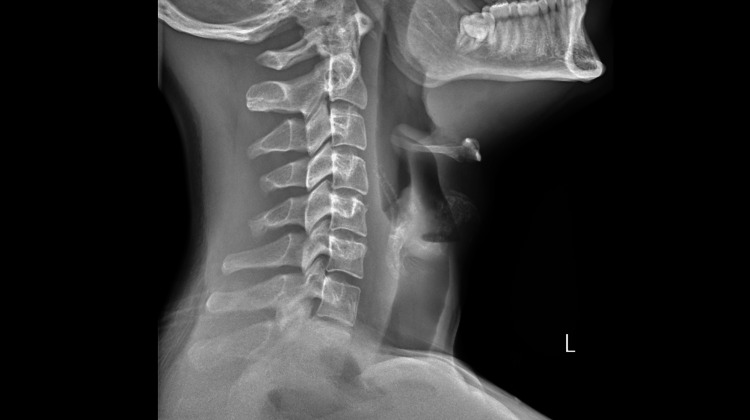
Lateral view neck radiograph showing swollen epiglottis

His vital signs were: heart rate of 118 beats per minute, blood pressure of 152/92 mm Hg, temperature 39.1^o^C, respiratory rate of 18 per minute, and saturation of 97% on room air. He described pain of 10 on a scale of 1 to 10, 10 being the highest. Laboratory analysis revealed a CRP of 185 mg/L and white cell counts of 29.7 (x10^9^/L) with a neutrophil count of 27.1 (x10^9^/L). Blood gases showed values within the normal range. A plain radiograph of a lateral view of the neck showed a swollen epiglottis and narrowing of the supra-glottic airway (Figure 1). The patient was initially diagnosed with tonsillitis in the emergency department as he had swollen bilateral tonsils and received a stat dose of intravenous Benzyl Penicillin and Metronidazole as per local guidelines.

An ENT specialist referral was made, and the on-call ENT doctor performed a bedside flexible nasopharyngoscopy, which revealed oedematous uvula and bilateral oedematous aryepiglottic folds. The glottic chink was adequate with normal and mobile bilateral vocal cords. A diagnosis of acute epiglottitis was made based on the above findings, and the patient was initially admitted to a high-dependency unit for close monitoring of his airway. He was started on daily Ceftriaxone intravenous antibiotic along with regular Dexamethasone to help decrease the airway oedema. Once he started improving clinically and his inflammatory markers improved as well, he was gradually stepped down to the ward, weaned off the antibiotics and steroids, and safely discharged home after four days of inpatient stay.

## Discussion

Adult epiglottitis, a rare yet potentially life-threatening condition, demands immediate recognition and management due to its propensity for rapid airway compromise. This condition's clinical presentation in adults can be insidious, often mimicking more common upper respiratory infections, which poses significant challenges in timely diagnosis and treatment.

The overall incidence of adult epiglottitis has been on a subtle rise, primarily attributed to the increasing recognition of non-Haemophilus influenzae aetiologies. Streptococcus pneumoniae, Staphylococcus aureus, and various viral agents are now more commonly identified as causative pathogens in adult cases [5]. This epidemiological shift away from H. influenzae is partly due to the successful implementation of the Hib vaccine, which has drastically reduced the disease burden in children.

Unlike in children, whose presentation is typically abrupt and dramatic, adults with epiglottitis may exhibit more gradual symptom onset [6]. Classical features like severe sore throat, fever, odynophagia, and voice changes, including a muffled or hoarse voice, are commonly reported [6]. Physical findings such as stridor, drooling, or respiratory distress signal impending airway obstruction and necessitate urgent intervention.

Imaging modalities like lateral neck radiographs can reveal the swollen "thumbprint" appearance of the epiglottis, although a CT scan provides more detailed information and helps rule out other causes of upper airway obstruction. Nonetheless, direct visualization through laryngoscopy, though risky, is definitive for diagnosis but should be reserved for controlled environments [7].

Securing the airway is the foremost priority in management, often requiring advanced airway interventions under experienced hands. Medical management typically involves broad-spectrum antibiotics, considering the polymicrobial nature of the infection. The empirical antibiotic therapy usually covers both gram-positive and gram-negative bacteria and anaerobes. The utility of corticosteroids in adult epiglottitis, mainly to alleviate oedema, is debated, although some clinicians use them as adjunct therapy [8].

If promptly recognized and treated, the prognosis of adult epiglottitis is generally favorable. However, delayed or inadequate management can lead to dire consequences, including acute airway obstruction, respiratory failure, and even death. Other complications like epiglottic abscess, cellulitis, and systemic spread of infection are also notable [9].

Preventive strategies, particularly in high-risk groups, and public health initiatives aimed at vaccine promotion against prevalent pathogens might reduce adult epiglottitis incidence. Furthermore, increasing awareness among healthcare providers regarding its atypical presentations in adults is crucial for early diagnosis and intervention [10].

## Conclusions

Adult epiglottitis, characterized by its subtle onset and potential for rapid progression to life-threatening airway obstruction, is a condition that requires a high index of suspicion and prompt, aggressive management. Understanding the evolving epidemiology, maintaining vigilance for atypical presentations, and readiness for emergency airway management is key to improving outcomes in adult epiglottitis.
